# Transient Potassium Channels: Therapeutic Targets for Brain Disorders

**DOI:** 10.3389/fncel.2019.00265

**Published:** 2019-06-13

**Authors:** Wonjun Noh, Sojeong Pak, Geunho Choi, Sungchil Yang, Sunggu Yang

**Affiliations:** ^1^Department of Nano-Bioengineering, Incheon National University, Incheon, South Korea; ^2^Department of Biomedical Sciences, City University of Hong Kong, Kowloon, Hong Kong; ^3^Department of Computer Science and Engineering, Incheon National University, Incheon, South Korea

**Keywords:** A-type potassium channels, NMDA receptor, VGCC, dendritic spikes, brain disorders

## Abstract

Transient potassium current channels (I_A_ channels), which are expressed in most brain areas, have a central role in modulating feedforward and feedback inhibition along the dendroaxonic axis. Loss of the modulatory channels is tightly associated with a number of brain diseases such as Alzheimer’s disease, epilepsy, fragile X syndrome (FXS), Parkinson’s disease, chronic pain, tinnitus, and ataxia. However, the functional significance of I_A_ channels in these diseases has so far been underestimated. In this review, we discuss the distribution and function of I_A_ channels. Particularly, we posit that downregulation of I_A_ channels results in neuronal (mostly dendritic) hyperexcitability accompanied by the imbalanced excitation and inhibition ratio in the brain’s networks, eventually causing the brain diseases. Finally, we propose a potential therapeutic target: the enhanced action of I_A_ channels to counteract Ca^2+^-permeable channels including NMDA receptors could be harnessed to restore dendritic excitability, leading to a balanced neuronal state.

## Introduction

Sequential synaptic inputs often cause dendritic spikes. Fast-acting membrane depolarization in the dendritic tree is converted into axo-somatic action potentials (APs) that do not only propagate down the axon but also back into the dendritic tree (Golding et al., 2001; Bender and Trussell, 2012). With distance from the soma, the amplitude of back-propagation action potentials (bAPs) is gradually attenuated by the inhibitory action of A-type transient potassium channels, I_A_ for short (Hoffman et al., 1997; Frick et al., 2003). It is because the density of these channels gradually increases from the soma to distal dendrites. In addition to the role of I_A_ on feedback propagation, the channels play a critical role in feedforward, pre-to-post-synaptic transmission and integration by suppressing dendritic spikes (Cai et al., 2004; Kim et al., 2007; Jung et al., 2008; Kim and Hoffman, 2008; Yang et al., 2015). Therefore, a dysfunction of I_A_ channels can lead to loss of control over neuronal excitability, the hallmark of several diseases in the brain. In this review, we discuss (1) the distribution (mostly in human) and function (in animals and human) of I_A_ channels in the brain, (2) the common down-regulation of I_A_ channels in several brain diseases and (3) a potential I_A_ channel-based therapeutic target.

## Subtypes of I_A_ Channels

The potassium channels form a large and diverse family of ion channels that are involved in establishing the resting membrane potential, determining the action potential waveform, regulating neurotransmitter release, and modulating rhythmic firing patterns (Llinas, 1988; Joho and Hurlock, 2009). There are four major classes of potassium channels in the brain: Calcium-activated, inwardly rectifying, leak, and voltage-gated potassium channel. Particularly, I_A_ channels, one type of voltage-gated potassium channels, are characterized by closed, opened, and inactivated channel states depending on the membrane voltage. I_A_ channels form a large macromolecular complex comprising four ion-conducting alpha-subunits and beta-subunits (either being cytoplasmic or transmembrane) with auxiliary regulatory and supporting proteins (Leicher et al., 1998; Grizel et al., 2014). This voltage-gated potassium channels usually have a homotetrameric structure (with all alpha-subunits being identical), but some of them are heterotetrameric (with two or more non-identical alpha-subunits) (Sokolova, 2004; Grizel et al., 2014). The alpha-subunits (Kv1.4, Kv3.3, Kv3.4, Kv4.1, Kv4.2, and Kv4.3) that are divided into discrete families on the basis of sequence similarity form an ion pore and infrastructure of the channel. The alpha-subunits determine the fast kinetic property of I_A_ channels, rapidly activating and inactivating (Coetzee et al., 1999; Covarrubias et al., 2008). The alpha-subunits are major components on which pharmacological agents target. Most generally used 4-aminopyridine (4-AP) not only blocks the subtypes of I_A_ channels such as Kv1.4 and Kv4.x, but also other subtypes of voltage-gated potassium channels (Gutman et al., 2005). Alternatively, biotoxins isolated from venoms of the tarantula spider (PaTx1 and 2, HmTx1, HpTx1 and 2), sea anemone (BDS I and II) and scorpion (alpha-KTx15 subfamily) have relatively higher selectivity on the specific subtypes of I_A_ channels (Sanguinetti et al., 1997; Diochot et al., 1999; Escoubas and Rash, 2004; Yeung et al., 2005; Prestipino et al., 2009). Plus, some components (Diclofenac and BmP02) have been identified to activate I_A_ channels (Liu et al., 2005; Wu et al., 2016a,b).

Meanwhile, the beta-subunits and other auxiliary subunits of I_A_ channels are known for modulating the biophysical properties and functions of I_A_ channels (Hoffman and Johnston, 1998; Coetzee et al., 1999; An et al., 2000; Anderson et al., 2000; Varga et al., 2000; Beck et al., 2002; Yuan et al., 2002; Nadal et al., 2003; Gebauer et al., 2004). The alpha-subunit in the Kv1 complex interacts with the amino-terminal tetramerization domain of Kv beta-proteins which regulates the gating of the channels (Wulff et al., 2009). The Kv3 complex contains potassium voltage-gated channel subfamily E regulatory subunit 3 (KCNE3) which is known to carry a fast inactivating current (Wulff et al., 2009). The Kv4 complex has been associated with various ancillary subunits or scaffolding proteins including Kv beta-subunits (Aimond et al., 2005), dipeptidyl peptidase (DPP) family members (DPP6 and DPP10) (Nadal et al., 2003; Jerng et al., 2005) and K^+^ channel-interacting proteins (KChIP1, KChIP2, KChIP3, and KChIP4) (An et al., 2000; Morohashi et al., 2002). In particular, the KChIPs are required for function and formation of the Kv4 complex (Marionneau et al., 2009) while DPPs and beta-subunits contribute to the alteration of Kv4 currents (Yang et al., 2001; Jerng et al., 2005).

The activity and expression of I_A_ channels can be modulated by certain post-translational modifications of phosphorylation and palmitoylation (Wulff et al., 2009) with various protein kinases such as Ca^2+^/calmodulin-dependent protein kinase II (CaMKII), cAMP-dependent protein kinase (PKA), protein kinase C (PKC), and mitogen-activated protein kinase (MAPK) (Jerng et al., 2004). CaMKII phosphorylation of Kv1.4 is reported to regulate the inactivation gate of I_A_ channels arising from Kv1.4 (Roeper et al., 1997; Varga et al., 2004). Similarly, CaMKII phosphorylated Kv4.2 upregulates both the expression level of Kv4.2 proteins and the peak current of Kv4.2 (Roeper et al., 1997; Varga et al., 2004). Meanwhile, either PKA or PKC activation decreases the opening of I_A_ channels encoded mostly by Kv4.2; accordingly, their activation increases the amplitude of bAPs in distal dendrites (Hoffman and Johnston, 1998). In other studies, PKC reduces inactivation of I_A_ channels encoded by Kv3.3 and Kv3.4 (Covarrubias et al., 1994). Thus, the complex structure of I_A_ channels likely determines their class and functions.

In this review, an attention is also made to (1) the microscopic expression pattern and (2) kinetics of Kv1.4 (*kcna4*), Kv3.3 (*kcnc3*), Kv3.4 (*kcnc4*), Kv4.1 (*kcnd1*), Kv4.2 (*kcnd2*), and Kv4.3 (*kcnd3*) which are the most common alpha-subunits of I_A_ channels (Coetzee et al., 1999; Covarrubias et al., 2008). Firstly, as for the I_A_ expression pattern in a neuron, pioneering works for characteristic I_A_ channels on the dendrites show that 4-AP (a non-selective I_A_ blocker)-sensitive I_A_ channels are distributed along the apical dendritic truck and tuft of L5B pyramidal neurons in mice (Bekkers, 2000; Korngreen and Sakmann, 2000; Harnett et al., 2013). Specifically, Kv1.4 and Kv3.4 channels are largely localized in axons and dendrites (Veh et al., 1995; Serodio and Rudy, 1998) while Kv3.3 is mostly located in distal dendrites (Veh et al., 1995; Kim et al., 2005; Chang et al., 2007). Also, Kv4 family such as Kv4.1, Kv4.2, Kv4.3 is primarily found in somatodendritic membrane although Kv4.2 channels are highly concentrated in dendrites (Coetzee et al., 1999; Rudy and McBain, 2001; Song, 2002; Kim et al., 2005; Kerti et al., 2012). Secondly, Kv3 subfamily (encoding Kv3.1, Kv3.2, Kv3.3, and Kv3.4) has distinct functional properties; they activate at high thresholds (-10 mV) with rapid kinetics (Rudy and McBain, 2001; Zhao et al., 2013). In fact, homomeric Kv3.1 and Kv3.2 channels have slower inactivation kinetics than heteromultimeric Kv3.1/Kv3.4 and Kv3.2/Kv3.4 channels carrying a fast inactivating component (Rudy and McBain, 2001). Also, Kv4 channels show the fast recovery from inactivation state, a hallmark of somatodendritic I_A_ channels. In general, I_A_ channels respond transiently to stimuli, exhibiting a rapidly activating and inactivating kinetic. They affect the excitability and firing properties of neurons via regulatory actions of their pore-forming alpha-subunits, each of which has slightly different sensitivity to voltage changes (Locke and Nerbonne, 1997; Carrasquillo et al., 2012).

## Close Association of I_A_ With Several Brain Diseases

**FIGURE 1:**
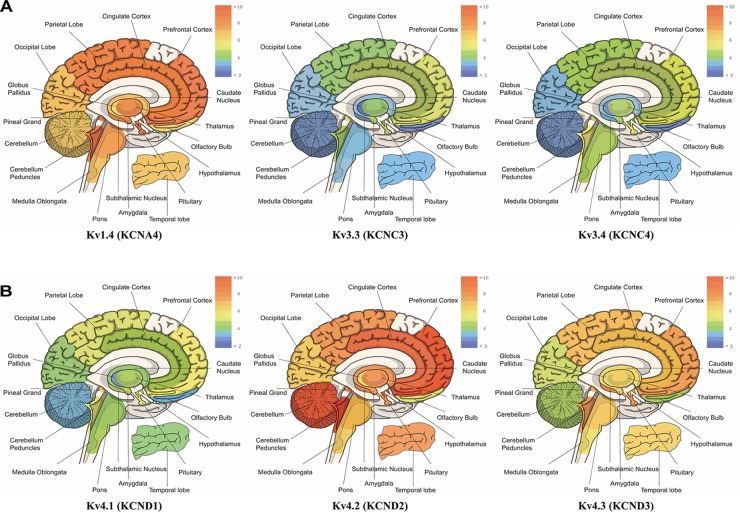
mRNA expression levels of panel **(A)** *kcna4* (Kv1.4), *kcnc3* (Kv3.3), and *kcnc4* (Kv3.4), panel **(B)** *kcnd1* (Kv4.1), *kcnd2* (Kv4.2), *and kcnd3* (Kv4.3). The microarray data have the mean value of each probe-set data obtained from BIOGPS (http://www.biogps.org), showing the tissue-specific pattern of mRNA expression according to GeneAtlas U133A, gcrma (Su et al., 2004). Note that expression of Kv1.4 is elevated in prefrontal cortex, cerebellum peduncles, pituitary and pineal gland; Kv3.3 in prefrontal cortex, pineal gland, pituitary, cerebellum peduncles; Kv3.4 in cerebellum peduncles, pituitary, prefrontal cortex and pineal gland; Kv4.1 in pineal gland, prefrontal cortex, pituitary and cerebellum peduncles; Kv4.2 in cerebellum peduncles, cerebellum, and prefrontal cortex and hypothalamus; Kv4.3 in subthalamic nucleus, pineal gland, cerebellum peduncles, pituitary and prefrontal cortex (when only top 25% of expression levels is considered).

Subtypes of I_A_ channels are differentially distributed in the brain. The macroscopic expression pattern of I_A_ channels is now investigated with human brain where clinical value can be highlighted. Kv1.4 is predominantly expressed in prefrontal cortex, cerebellum peduncles, pituitary and pineal gland in a ranking order; kv3.3 in prefrontal cortex, pineal gland, pituitary and cerebellum peduncles; kv3.4 in cerebellum peduncles, pituitary, prefrontal cortex and pineal gland; kv4.1 in pineal gland, prefrontal cortex, pituitary and cerebellum peduncles; kv4.2 in cerebellum peduncles, cerebellum, prefrontal cortex and hypothalamus; kv4.3 in subthalamic nucleus, pineal gland, cerebellum peduncles and prefrontal cortex (Figure 1 and Table 1). Furthermore, there are significant functional consequences of I_A_ on brain disorders (Table 2). Interestingly, the mRNA expression of I_A_ channels is largely downregulated in the condition of brain dysfunction (Table 3). In the following sections, we discuss the tight involvement of I_A_ channels in brain diseases, such as Alzheimer’s disease, epilepsy, fragile X syndrome (FXS), Parkinson’s disease, chronic pain, tinnitus and ataxia.

**Table 1:** mRNA expression levels of each I_A_ channel subunit on the human brain.

	mRNA Expression Level					
	
	KCNA 4 (Kv1.4)	KCNC 3 (Kv3.3)	KCNC 4 (Kv3.4)	KCND 1 (Kv4.1)	KCND 2 (Kv4.2)	KCND 3 (Kv4.3)
Olfactory bulb	6.1	3.2	3.3	3.7	5.65	4.9
Globus pallidus	5.95	2.95	3.1	3.4	5.85	5.15
Occipital lobe	7.15	3.7	3.85	4.2	6.55	5.525
Medulla oblongata	7.05	3.8	4.9	4.3	6.6	5.875
Pons	7.75	3.9	4.8	4.45	7.35	6.75
Subthalamic nucleus	7.45	3.65	3.9	5.05	7.55	7.9
Amygdala	8.3	4.4	4.45	4.95	7.9	6.125
Caudate nucleus	7.1	3.7	3.8	4.15	8.3	6.1
Parietal lobe	8.4	4.25	4.6	5	8.4	6.925
Thalamus	8.05	4.1	4.35	4.75	8.55	6.6
Pituitary	9.65	4.75	5.5	5.45	8.65	7.325
Temporal lobe	7.2	3.75	3.85	4.25	8.65	6.675
Pineal grand	9.88	5.05	5.11	6.36	8.74	7.465
Cingulate cortex	8.1	4.2	4.3	4.8	9.4	6.75
Hypothalamus	7.95	4.5	4.45	5.1	10.75	6.5
Prefrontal cortex	9.2	5.1	5.2	5.75	13.65	7.325
Cerebellum	6.4	3.45	3.4	3.85	21.05	5.15
Cerebellum peduncles	9	4.6	5.55	5.35	58.2	7.4


*The microarray data have the mean value of each probe-set data obtained from BIOGPS (http://www.biogps.org), showing the tissue-specific pattern of mRNA expression according to GeneAtlas U133A, gcrma (Su et al., 2004): KCNA4 (http://ds.biogps.org/dataset=GSE1133&gene=3739); KCNC3 (http://ds.biogps.org/dataset=GSE1133&gene=3748); KCNC4 (http://ds.biogps.org/?dataset=GSE1133&gene=3749); KCND1 (http://ds.biogps.org/?dataset=GSE1133&gene=3750); KCND2 (http://ds.biogps.org/?dataset=GSE1133&gene=3751); KCND3 (http://ds.biogps.org/?dataset=GSE1133&gene=3752).*

**Table 2:** The close association of I_A_ channel activity and expression in brain diseases.

Brain Disease	Subject	Regions	Central Findings	References
Alzheimer’s	mouse CA1	Hippocampus (CA1)	A_β_ reduces I_A_ conductance; reduced I_A_ current causes increased Ca2+ influx and excitotoxicity.	Morse et al., 2010
	SD rats (7–14 weeks)		A_β_ causes loss of Ca2+ regulation by inhibiting I_A_ channels in hippocampal CA1 pyramidal neurons, leading to functional and/or structural synaptic deficits in hippocampus.	Chen, 2005
	mouse (3 months)		A_β_-induced dendritic hyperexcitability is associated with the depletion of Kv4.2 and abnormalities in both EEG (electroencephalography) and behavior.	Hall et al., 2015
Epilepsy	Wistar rats (adult male)	Hippocampus	The expression of I_A_ channel subunit Kv4.2 is selectively reduced in ischemic rats with spontaneous behavioral seizures and increased seizure susceptibility.	Lei et al., 2016
	mouse (12 and 30 weeks)		Kv 4.2 deficiency contributes to aberrant network excitability and increased seizure susceptibility.	Barnwell et al., 2009
	SD rat	Hippocampus (CA1)	Lack of I_A_ channels contributes to increased excitability and lowered seizure thresholds.	Castro et al., 2001
		Hippocampus	Altered phosphorylation and localization of Kv4.2 in status epilepticus.	Lugo et al., 2008
	human	Temporal lobe (Amygdala/hippocampus)	Temporal lobe epilepsy patient carries an I_A_ channel gene mutation, namely a Kv4.2 truncation mutation. This mutation causes aberrant neuronal excitability, characteristic of temporal lobe epilepsy.	Singh et al., 2006
Fragile X	*Fmr1* K.O mouse (3 and 8 weeks)	Hippocampus (CA1 and dentate gyrus)	Loss of FMRP expression causes the dendritic downregulation of Kv4.2 channels, possibly leading to neuronal hyperexcitability.	Gross et al., 2011
	*Fmr1* K.O mouse (5–7 weeks)	Hippocampus (CA1)	Functional Kv4.× channels in the dendrites of CA1 pyramidal neurons are downregulated.	Routh et al., 2013
	*Fmr1* K.O mouse (Varied age)		A lack of FMRP decreased Kv4.2 expression in dendrite, but the Kv4.2 was restored with NMDA treatment.	Lee et al., 2011
Tinnitus	hearing loss-induced tinnitus animal model (3 months)	Temporal Lobe (Auditory Cortex)	Downregulation of Kv1.4 gene expression in auditory cortex in a noise-induced animal model of tinnitus.	Tetteh et al., 2017
Parkinson’s	A53T-SNCA mice (7–8 months)	Brainstem SN/VTA	A dysfunction of Kv 4.3 channels increases intrinsic firing rates of DA SN neurons in part by increasing their intrinsic pacemaker frequency.	Subramaniam et al., 2014
Chronic Pain	CD-1 mouse, 129SvEv, Kv4.2^-^/^-^ model (4–10 days)	Cultured/Sliced Spinal Cord DH neurons	Kv4.2 knockout in mice up-regulates hypersensitivity to tactile and thermal stimuli.	Hu et al., 2006
	SD rats, Cystitis model (170–220 g)	DRG neuron	Reduction of I_A_ contributes to hyperexcitability of capsaicin-sensitive C-fiber bladder afferent neurons in rats with HCl-induced cystitis.	Hayashi et al., 2009
	SD rats, diabetic neuropathic model (9 weeks old)		Reduction of I_A_ in primary sensory neurons in a rat diabetic neuropathy model.	Cao et al., 2010
	SD rats, neuropathic pain model (250–300 g)		Protein expression of Kv4.3 in DRG neurons is decreased in a rat neuropathic pain model, leading to mechanical hypersensitivity.	Chien et al., 2007
	C57BL/6J mouse, neuropathic pain model (20–25 g)		Peripheral nerve injury decreases the mRNA level of Kv4.3 expression.	Uchida et al., 2010
Ataxia	HEK293T cells	HEK293T cells	KCND3 mutations cause SCA19 by impaired protein maturation and/or reduced channel function	Duarri et al., 2013
			Autosomal dominant cerebellar ataxia (SCA type 22) shows heterozygous mutations in the voltage-gated potassium channel Kv4.3-encording KCND3 gene.	Lee et al., 2012
	CHO cells	CHO cells	Kv3.3 gene mutations on chromosome 19q13 cause the neurological disorder SCA type 13.	Zhao et al., 2013
	Filipino for cerebellar Ataxia	*Xenopus laevis* oocytes	Mutation associated with SCA type 13 alter KCNC3 function and expression in expression systems of *Xenopus laevis* which were injected by Filipino-cerebellar ataxia cDNA clone.	Waters et al., 2006


*Ab, amyloid beta; SD, sprague dowley; FMRP, fragile X mental retardation protein; SN, substantia nigra; VTA, ventral tegmental area; DRG, dorsal root ganglion; DH, dorsal horn.*

**Table 3:** Effects of I_A_ channel mRNA expression in brain diseases.

Brain Diseases	Subject	Region	Genetic Basis of I_A_ Channel	*P*-Value Control vs. Brain Diseases	References
Alzheimer’s disease	Human patient	Temporal lobe	KCNA4_1	0.099	GSE6834
			KCNA4_2	0.086	
			KCNA4_3	0.078	
	Human patient	Cerebellum	KCNA4_1	0.070	
			KCNC3_1	0.081	
			KCND2_1	0.072	
	Human patient	Frontal cortex	KCND2_1	0.071	GSE36980
		Temporal cortex	KCND2_1	0.006^**^	
			KCND3_1	0.043^*^	
		Hippocampus	KCNA4_1	0.081	
			KCND2_1	0.079	
			KCND3_1	0.0002^**^	
Parkinson’s disease	Human post-mortem	Putamen (basal ganglia)	KCNA4_1	0.040^*^	GSE20291
			KCNC3_1	0.038^*^	
			KCNC4_1	0.050	
			KCND1_1	0.068	
			KCND2_1	0.026^*^	
			KCND3_1	0.029^*^	
			KCND3_2	0.034^*^	
			KCND3_3	0.044^*^	
			KCND3_4	0.076	
		Prefrontal area 9	KCND2_1	0.058	GSE20168
			KCND3_1	0.012^*^	
Epilepsy	Human patient	Temporal lobe	KCNC3_1	0.0002^**^	GSE6834
			KCNC3_2	0.0004^**^	
			KCNC3_3	0.004^**^	
Epilepsy	Human patient	Temporal lobe	KCNC3_4	0.048^*^	GSE6834
			KCNC4_1	0.030^*^	
			KCNC4_2	0.059	
			KCND2_1	0.102	
			KCND3_1	0.008^**^	
			KCND3_2	0.011^*^	
Epilepsy (Febrile seizures)	Human patient	Hippocampal CA3	KCNA4_1	0.001^**^	GSE28674
			KCNC3_1	0.002^**^	
			KCND2_1	0.024^*^	
			KCND2_2	0.029^*^	
Fragile X syndrome	Mouse (4, 8, 12 weeks)	Cerebellar purkinje cells	KCNA4_1	0.071	GSE57034
			KCND2_1	0.064	
			KCND3_1	0.040^*^	
	Embryo mouse (17–18 days)	Cortex	KCNC3_1	0.045^*^	GSE71034
			KCNC4_1	0.058	
			KCND3_1	0.002^**^	
		Cortex primary neuron	KCNA4_1	0.017^*^	
			KCNA4_2	0.006^**^	
			KCND2_1	0.030^*^	
			KCND2_2	0.022^*^	
			KCND3_1	0.020^*^	
		Hippocampus primary neurons	KCND2_1	0.074	
			KCND3_1	0.072	
Tinnitus	Mouse (3 months)	Auditory cortex	KCNA4_1	0.042^*^	65
Chronic pain	C57BL/6 X CBA/J mouse (Adult male)	Trigeminal ganglia	KCNC3_1	0.011^*^	GSE69619
			KCND1_1	0.016^*^	
			KCND2_1	0.063	
			KCND2_2	0.02^*^	
Chronic pain	C57BL/6 X CBA/J mouse (Adult male)	Trigeminal ganglia	KCND2_3	0.009^**^	GSE69619
			KCND2_4	0.067	
			KCND3_1	0.078	
Ataxia	Human (Normal vs. Ataxia)	Cerebellum	KCND2_1	0.000000007^**^	GSE61019
			KCND3_1	0.015^*^	
	C57BL/6 mouse (6 weeks)		KCNC4_1	0.046^*^	GSE61908
			KCND1_1	0.048^*^	
	C57BL/6 mouse (6 weeks)		KCND2_1	0.043^*^	
			KCND3_1	0.042^*^	
	C57BL/6 mouse (24 months)		KCNC3_1	0.061	GSE55177
			KCND1_1	0.032^*^	
			KCND2_1	0.08	
			KCND3_1	0.009^**^	
	C57BL/6 mouse (12 months)		KCNC3_1	0.0000007^**^	
			KCNC3_2	0.00005^**^	
			KCNC3_3	0.011^*^	
			KCNC4_1	0.007^**^	
			KCNC4_2	0.007^**^	
			KCND2_1	0.079	
			KCND3_1	0.007^**^	
			KCND3_2	0.022^*^	
			KCND3_3	0.035^*^	
	C57BL/6 mouse (6 months)		KCNA4_1	0.063	
			KCNC3_1	0.002^**^	
			KCNC4_1	0.043^*^	
			KCND3_1	0.068	
			KCND3_2	0.072	
			KCND3_3	0.095	


*We analyzed human and animal gene expression data from GEO (http://www.ncbi.nlm.nih.gov/geo). Data were analyzed by GEO2R (http://www.ncbi.nlm.nih.gov/geo/geo2r/). GSE6834 and GSE36980 were analyzed for Alzheimer’s disease. GSE20168 and GSE20291 were analyzed for Parkinson’s disease. GSE6834 and GSE28674 were analyzed for epilepsy, GSE57034 and GSE71034 were analyzed for fragile X syndrome. GSE69619 was analyzed for Chronic pain. GSE61019, GSE55177, and GSE61908 were analyzed for Ataxia. Tinnitus data was analyzed by Affymetrix. Statistical significance, ^∗^*p* < 0.05; ^∗∗^*p* < 0.01.*

## Alzheimer’s Disease

Alzheimer disease (AD) is a neurodegenerative disease characterized by progressive deterioration of cognitive function. The early symptoms of AD are found in language, perception, movement, and memory. In the final phase, it causes widespread neuronal death, leading to severe memory loss, emotional disturbance and language dysfunction. Thus, patients’ independent living is impossible and relies on caregivers (Chen, 2005). One potential cause of AD appears to be the abnormal folding of the proteins Tau and/or Amyloid beta (A_β_) (Angulo et al., 2004). Interestingly, I_A_ channels are downregulated with the plaque of Tau and/or Amyloid beta (A_β_), as reported in a study of cultured hippocampal neurons from an AD animal model (Hokama et al., 2014). This is further evidenced by other studies that A_β_-induced dendritic hyperexcitability is known to arise from a sustained increase in intracellular Ca^2+^ and dysfunction of I_A_ channels in dendrites on hippocampal CA1 neurons of rodents (Chen, 2005; Morse et al., 2010; Hall et al., 2015). The loss of Ca^2+^ homeostasis in dendrites is attributed to abnormally enhanced back-propagation action potentials (bAPs) resulting from dysfunction of I_A_ channels. In other words, the dysfunction of I_A_ channels induced by A_β_ misfolding and aggregation results in excessive dendritic Ca^2+^ influx, leading to the subsequent destruction of synapses and ultimately cell death. In contrast, a study reported that up-regulation of the kv3.4 channel (but not caused by A_β_ deposition) causes alterations of the neuron activity in early AD (Angulo et al., 2004). In any cases, it is likely that the alteration of I_A_ channels disrupts synaptic function of neurons, being involved in the clinical manifestation of AD.

## Epilepsy

Epilepsy is characterized by repeated convulsive seizures. Seizures are known to be caused by excessive excitation or suppressed inhibition in neural networks (Yang and Cox, 2008, 2011; Park et al., 2018). One-third of epilepsy is a generalized seizure often accompanied by loss of consciousness, affecting the entire brain. Two-thirds of epilepsy occurs as focal seizures. Then, it often proceeds into the generalized seizures (Appleton et al., 2012). Epileptic seizure occurs as the result of environmental factors such as brain injury, stroke, brain tumors and infections, and genetic factors. Also, many of the patients are often accompanied by psychological symptoms such as anxiety and depression (Engel, 2001; Vadlamudi et al., 2003; Berkovic et al., 2006). In any case, epileptic seizures are strongly associated with the downregulation of I_A_ channels (Tables 2, 3). In fact, the drugs that downregulate the activity of I_A_ channels have been recognized as pro-convulsants and, therefore, inhibiting the activity and expression of I_A_ channels is the well-established models of epilepsy (Bernard et al., 2004; Su et al., 2008). Indeed, in adult Wistar rats with spontaneous behavioral seizures, the expression of Kv4.2 is decreased in the hippocampus (Lei et al., 2016). Also, in a seizure induction study, pregnant rats were infused with a neurotoxin, MAM (methylazoxymethanol). This neurotoxin reduced DNA synthesis and caused neuronal heterotopia in the hippocampus of the rat fetuses. Postnatally, their hippocampal neurons showed I_A_ channels (encoded by Kv4.2) dysfunctional and exhibited hyperexcitability. As a result, their seizure thresholds were lower than those in normal rats (Castro et al., 2001). With status epilepticus induced by kainic acid, the rat model is associated with the activation of extracellular signal-regulated kinase (ERK), an enzyme that causes the structural and functional impairment of dendritic Kv4.2-I_A_ channels (Lugo et al., 2008). Apart from the expression level change of Kv4.2 in animal seizure model, the hippocampus of Kv4.2 knockout (KO) mice increases the sensitivity to convulsion-inducing stimulation compared to that in wild type mice, indicating the depletion of Kv4.2 is associated with deviant network excitability and increased seizure susceptibility (Barnwell et al., 2009). Consistent with these findings in animals, a study with surgical tissue from patients with hippocampal sclerosis-induced epilepsy showed that the dendritic regions of the hippocampus exhibit the decreased expression of Kv4.2. Similarly, the patient with one temporal lobe epilepsy carried a gene mutation of Kv4.2 channels (Singh et al., 2006; Aronica et al., 2009). Thus, these results suggest that the functional and/or structural reduction of I_A_ channels is associated with abnormal neuronal excitability and epilepsy in the temporal lobe in both animals and humans.

## Fragile X Syndrome

Fragile X syndrome (FXS) is the most common heritable mental disability. In general, the person with FXS has a long, narrow face, big ears and flexible fingers, often having autistic behaviors. FXS is associated with the dysfunction of the protein *fragile X mental retardation protein* (FMRP) encoded by a gene FMR1 on the X chromosome, a regulator of protein synthesis in axons and dendrites (Deng et al., 2011). FMRP possessing multiple RNA binding domains binds to polyribosome complexes and regulates protein synthesis (Ashley et al., 1993; Siomi et al., 1993; Brown et al., 2001; Greenough et al., 2001; Li et al., 2001; Todd et al., 2003; Darnell et al., 2005). Loss of FMRP causes a variety of symptoms such as sensory hypersensitivity and repetitive/excessive behavior which are hallmarks of FXS (Musumeci et al., 1999; Bureau et al., 2008; Bhakar et al., 2012; Yang et al., 2014b; Zhang et al., 2014). Recent findings show that FMRP is associated with the expression and activation of Kv4.2 channels in the mouse hippocampus (Table 2). There are two different conclusions on the interactional relation between FMRP and Kv4.2 channels. On one hand, in an *fmr1* KO mouse model, the dendritic protein level of Kv4.2 in the hippocampus was found to be reduced, possibly causing neural hyperexcitability which underlies a plausible mechanism of FXS (and its associated epilepsy) (Gross et al., 2011). Also, the finding is supported by a following study with *fmr1* KO mice that Kv4.x-mediated currents are reduced in dendrites, but not soma, of hippocampal CA1 pyramidal neurons, and accordingly the amplitude of back-propagation action potentials (bAPs) is increased in distal dendrites (Routh et al., 2013). On the sharp contrary, the local translation of Kv4.2 in CA1 dendrites increases in *fmr1* KO mice, indicating that FMRP is a negative controller of dendritic Kv4.2 (Lee et al., 2011). In any cases, dendritic I_A_ channels have been found important in FXS. Further studies are required to explore the dynamic role of dendritic I_A_ channels in FXS.

## Parkinson’s Disease

Parkinson’s disease (PD) is one of the most common neurodegenerative disorders characterized by an impairment of dopaminergic systems. Patients who suffer from PD have problems with voluntary behavior and thinking. Dementia and emotional problems such as depression and anxiety can be gradually developed over time. The cause of PD has several candidates: (1) environmental factors such as exposure to pesticides and a history of head injury and (2) genetic factors related to certain genes including SNCA, LRRK2, GNA, PRKN, PINK1, PARK7, VPS35, EIF4G1, DNAJC13, and CHCHD2 (Kalia and Lang, 2015). The pathological hallmark of PD is known to be the loss of dopaminergic neurons in the substantia nigra (SN). Another pathological feature of PD is the formation of protein aggregates called Lewy bodies (Davie, 2008). Furthermore, PD is also associated with dysfunction of I_A_ channels (Lawson, 2000). For instance, the inhibition of Kv4.3 channels increases the spontaneous activity of nigral dopamine (DA) neurons and disrupts DA release, ultimately inducing the onset of PD (Subramaniam et al., 2014). In the research, the enhanced neuronal firing was observed only in DA neurons of the SN, but not in DA neurons of the ventral tegmental area. These animal studies are consistent with findings that I_A_ expression is altered in PD patients, as shown in Table 3.

## Chronic Pain

Chronic pain severely affects most daily activities, disrupting social life with financial burden (Breivik et al., 2006; Bouhassira et al., 2008; Henschke et al., 2015). Two symptoms dominate the disease: (1) inflammatory pain caused by a tissue reaction to injury or infection and (2) neuropathic pain from nerve injury, which prevails after the primary damage has healed (Woolf and Salter, 2000). At the root of chronic pain lies the activity of sensory neurons in the dorsal root ganglion (DRG) and spinal dorsal horn (DH). These neurons are responsible for transmitting nociceptive information from the periphery to the brain. Chronic pain is largely associated with neuronal hyperexcitability of the DRGs and DH neurons. Interestingly, several lines of evidence indicate that these neurons’ hyperexcitability is caused by reduced inhibitory currents and/or expression of I_A_ channels in the DRGs and DH neurons (see Table 2). For example, in a rat diabetic neuropathy model, the reduction of 4-aminopyridine (4-AP)-sensitive I_A_ currents and the decreased mRNA expression level of I_A_ alpha-subunits; Kv1.4, Kv4.2, and Kv4.3 in the medium and large-diameter DRG neurons leads to the hyperexcitability of the neurons through BDNF activity on TrkB receptor (Cao et al., 2010). In addition, by peripheral nerve injury resulting in neuropathic pain, hypoacetylated histone H4 at the location of Kv4.2-NRSE is associated with the decreased mRNA expression of Kv4.3 in mouse DRG neurons, and the reduction of Kv4.3 is implicated in neuronal hyperexcitability (Kim et al., 2002; Uchida et al., 2010). Finally, DRGs in a neuropathic rat model show reduced Kv4.3 protein expression after spinal nerve ligation to DRGs, which drives mechanical hypersensitivity (Chien et al., 2007). Apart from DRGs, neurons in the DH of animals with formalin-induced inflammatory chronic pain are Kv4.2-deficient and exhibit increased excitability, resulting in increased sensitivity to tactile and thermal stimuli (Hu et al., 2006). Moreover, in a rat model of cystitis with visceral hypersensitivity, the decrease in 4-aminopyridine (4-AP)-sensitive I_A_ current as well as the decreased expression of Kv1.4 channels are observed in DH neurons, leading to neuronal hyperexcitability (Hayashi et al., 2009). In fact, the role of I_A_ channels in chronic pain has so far been studied mostly in the DRGs and DH neurons. It would be of great interest to investigate the role of I_A_ channels along the somatosensory pathway.

## Tinnitus

Tinnitus is a ringing in the ear that can be perceived even in the absence of external acoustic stimuli. Tinnitus is induced mainly by environmental factors such as traumatic noise, aging and ear infection. Environmental damage to cochlear hair cells alters the activity of the neural networks along the ascending auditory pathway, increasing the ratio of excitation to inhibition in central auditory neurons. Our group previously reported that auditory neurons in animals with noise-induced tinnitus exhibit intrinsic hyperexcitability. Also, in our preliminary data, noise-induced tinnitus is probably associated with the downregulation of Kv1.4 channels in rat auditory neurons (Yang et al., 2012; Tetteh et al., 2017). Thus, we believe this hyperexcitability to be due to the abnormal expression and/or function of I_A_ channels (Table 2).

## Ataxia

Ataxia is a neurodegenerative disorder caused by neural atrophy along the cerebellum-spine axis. Patients suffer from movement discoordination such as progressive ataxic gait and limb movements as well as difficulties with speech and eye movements (Duarri et al., 2013). Ataxia has three types depending on the parts of the dysfunction: (1) cerebellar ataxia due to dysfunction of the cerebellum; (2) sensory ataxia due to dysfunction of the sensory system including dorsal columns of the spinal cord, thalamus and parietal lobes; (3) vestibular ataxia due to dysfunction of the vestibular system. The main causes of ataxia are the focal lesion of corresponding CNS region, the exogenous substance like ethanol and vitamin b12 deficiency. Besides, genetic factors affect the degeneration of the cerebellum and/or the spine; 22 genes related to spinocerebellar ataxia (SCA) have been identified so far (Durr, 2010; Sailer and Houlden, 2012). The mRNA of Kv4.3 channels is highly expressed in the cerebellum of humans (Figure 1). Indeed, in animals and human patients with ataxia (Table 3), Kv4.3 channel mRNA expression is altered. For example, SCA type 19 and 22 are caused by mutation of Kv4.3 gene (Duarri et al., 2012, 2015; Lee et al., 2012; Table 2). In addition to Kv4.2 channels, Kv3.3 is the causative gene of SCA type 13, an autosomal dominant neurological disorder (Zhao et al., 2013). There are lines of evidence that SCA type 13 is caused by point mutations in the coding region of the Kv3.3 gene on chromosome 19q13 (Herman-Bert et al., 2000; Waters et al., 2005, 2006; Zhao et al., 2013). Moreover, the expression and function of Kv3.3 channels are altered in CHO cells transfected with mutated SCA13 as well as mRNA expression of Kv3.3 in animal with cerebellar ataxia (Herman-Bert et al., 2000; Waters et al., 2005, 2006; Zhao et al., 2013); Table 3). Taken all together, the alteration of I_A_ channels is deeply involved in several types of ataxia.

## Cellular Mechanisms and Therapeutic Targets of I_A_

**FIGURE 2:**
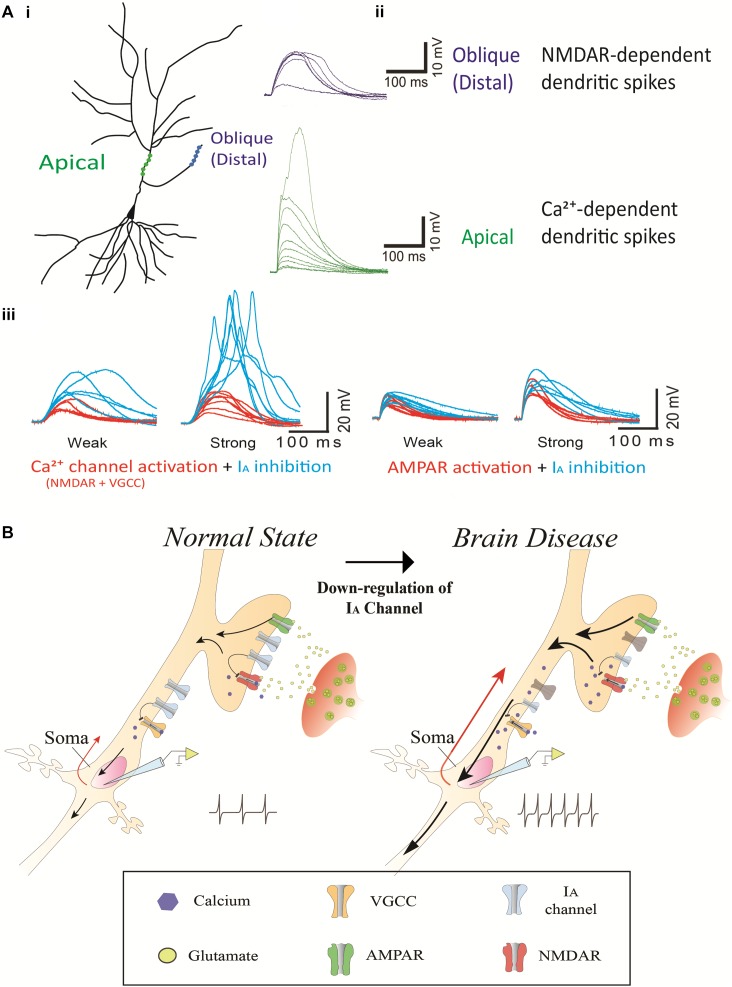
The contribution of I_A_ channels on neuron excitability and brain disease. **(Ai)** A schematic of two photoactivated locations (distal and apical trunk) of a hippocampal neuron. **(Aii)** Examplar gluEPSPs (or glutamate-evoked EPSPs) in response to photoactivation in the distal and apical trunk. Note that there is a sudden increase in the response, being referred to as a NMDA-dependent dendritic spike and Ca^2+^-dependent dendritic spike in the distal and apical trunk, respectively. **(Aiii)** The blockage of 4-AP (3 mM) sensitive-I_A_ channels potentiates the responses of NMDARs but not AMPARs. The left traces illustrate NMDAR (and VGCC)-mediated EPSPs, respectively, in a thin oblique dendrite or in an apical dendrite over an input strength; weak (averaged *6.3 μJ*) and strong (averaged *11.2 μJ*) energy. The right traces illustrate AMPAR-mediated EPSPs in a thin oblique dendrite over an input strength; weak (averaged *2.4 μJ*) and strong (averaged *4.3 μJ*) energy. **(B)** A putative model regarding the contribution of I_A_ channels on neuron excitability and brain disease. It shows a molecular mechanism that dendritic impairment of I_A_ channels can augment AP generation. The data was reused with permission from Yang et al. (2015).

I_A_ channels and Ca^2+^-permeable channels including NMDA receptors (NMDARs) and voltage-gated calcium channels (VGCCs) are abundant in dendrites, and their interaction determines the level of dendritic excitability and synaptic plasticity (Frick et al., 2003; Cai et al., 2004; Losonczy and Magee, 2006; Kim et al., 2007; Jung et al., 2008; Losonczy et al., 2008; Wang et al., 2014; Yang et al., 2015). Ca^2+^ channels are kinetically slow to activate and inactivate (Figure 2A; Yang et al., 2015). Ca^2+^ dendritic spikes are regenerative and, thus, difficult to regulate once initiated. To efficiently suppress Ca^2+^-mediated hyperexcitability, an active filter is required just before it occurs. Rapid action of I_A_ channels may play such a role as the active filter. For instance, NMDA spikes are mostly induced in distal dendrites of cortical neurons where most synaptic events occur (Larkum et al., 2009; Yang et al., 2011, 2014a, 2016). In turn, VGCCs magnify the NMDA-mediated dendritic spikes in apical dendrites, propagating the spikes to the axo-somatic sodium spikes zone for AP generation. Here, I_A_ channels could suppress the anterograde propagation of local Na^+^- and Ca^2+^-mediated signals from dendrites as well as the backward spread of APs into dendrites (Losonczy and Magee, 2006; Losonczy et al., 2008). When I_A_ channels are not active, the conductance of Ca^2+^-permeable channels can easily cause the membrane to slip toward depolarization, leading to synaptic enhancement in the axon terminals. In fact, internalization-induced reduction of I_A_ channels enhances synaptic plasticity while increased I_A_ channels do not display synaptic plasticity, suggesting a synaptic role of Ca^2+^-permeable channels heavily relying on the activity of I_A_ channels (Kim et al., 2007; Jung et al., 2008). The activity of Ca^2+^-permeable channels which are inflated by inactive I_A_ channels can often cause excessive neuronal excitability, resulting in an imbalance of excitation and inhibition in the whole network. We view this mechanism as a common cause for brain diseases such as Alzheimer’s disease, epilepsy, FXS, Parkinson’s disease, chronic pain, tinnitus and ataxia (Figure 2B).

Most drugs that are currently used to treat these diseases directly modulate ion channels and receptors that affect intrinsic and synaptic properties. However, as most of these ion channel-related drugs act across the whole brain, they produce a wide range of non-specific side effects, leading to the discontinuation of many potential treatments in the past. For instance, antagonists of NMDA receptors and voltage-sensitive sodium channels could in principle be used to suppress hyperexcitability. However, they also disrupt the physiological function of unintended brain areas. Furthermore, the drugs that activate GABA receptors also suppress other brain areas, often resulting in sedating and over-suppression effects that significantly reduce the quality of life. One way to circumvent these limitations of classical excitatory or inhibitory drugs would be to develop chemical compounds that “indirectly” modulate Na^+^ channels, NMDA or GABA receptors. A recent study reports that the application of 4-AP restores low- and high-threshold dendritic spikes in the distal and apical dendrites, respectively, which can be driven by Ca^2+^-permeable channels (NMDARs and/or Ni^2+^-sensitive voltage-gated Ca^2+^ channels) in rat hippocampal CA1 neurons (Yang et al., 2015). A similar study demonstrates that glutamate-mediated dendritic spikes are enhanced by 4-AP treatment and normalized by D-AP5 (an NMDA receptor antagonist) in mouse L3 pyramidal neuron (Biro et al., 2018). Thus, Ca^2+^-permeable channels and I_A_ channels play a counterbalancing role in regulating neuronal excitability (Figure 2). In this respect, I_A_ channels as an indirect modulator can be predominantly activated in a hyperexcitable condition caused by excessive activity of Ca^2+^-permeable channels, yet having minimal impact on the resting condition of neurons. Furthermore, given homeostatic regulation of neural networks, the lack of sensory or modulatory inputs likely causes the increment of excitation and/or decrement of inhibition in the affected neurons. These changes seem to be required for maintaining network excitability for neuron survival. In the hyperexcitable condition of the affected neurons often causing brain disorders, for example, an agonist of I_A_ channels can function to modulate neuronal excitability by counteracting against Ca^2+^-permeable channels/receptors. Thus, I_A_ channels as a therapeutic target may open an avenue for more sustainable treatments of brain disease with minimal interference in normal physiological function, leading to long-term clinical benefits. However, it is notable that there are limitations incurred when attempting to link the animal disease model to human neuropathology in the context of the expression and function of I_A_ channels. It is currently unclear whether the suggested therapeutic mechanism of I_A_ channels with animal disease models can apply to human diseases having a different neural network. Further research is required to delineate the precise contributions of I_A_ channels to brain diseases in human.

## Author Contributions

SgY and ScY designed, conceived, and wrote the manuscript. WN, GC, and SP collected the data, carried out data analysis, and prepared the manuscript. All authors reviewed the manuscript.

## Conflict of Interest Statement

The authors declare that the research was conducted in the absence of any commercial or financial relationships that could be construed as a potential conflict of interest.
